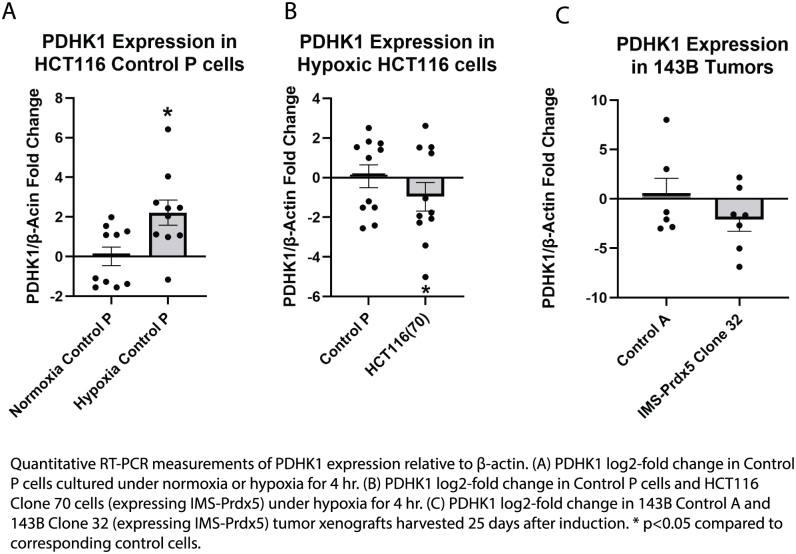# H_2_O_2_ transit through the mitochondrial intermembrane space promotes tumor cell growth *in vitro* and *in vivo*

**DOI:** 10.1016/j.jbc.2023.104624

**Published:** 2023-03-18

**Authors:** Simran S. Sabharwal, V. Joseph Dudley, Charlène Landwerlin, Paul T. Schumacker

**Affiliations:** 1 Department of Pediatrics, Feinberg School of Medicine, Northwestern University, Chicago, Illinois, USA; 2 Stanley Manne Children’s Research Institute of the Ann & Robert H. Lurie Children’s Hospital of Chicago, Chicago, Illinois, USA

**Keywords:** reactive oxygen species, redox signaling, hypoxia-inducible factor-1, mitochondrial intermembrane space, peroxiredoxin-5, cancer

## Abstract

Cancer cells experience increased levels of oxidant stress as a consequence of oncogene activation, nucleotide biosynthesis, and growth factor receptor signaling. Mitochondria contribute to this redox stress by generating reactive oxygen species (ROS) along the electron transport chain, which are released to the matrix and the intermembrane space (IMS). Assessing the contribution of mitochondrial ROS in cancer cells is technically difficult, as electron transport chain inhibitors can increase or decrease ROS generation, while they also block oxidative phosphorylation and ATP synthesis. Mitochondria-targeted antioxidant compounds can scavenge ROS in the matrix compartment but do not act on ROS released to the IMS. We assessed the importance of mitochondrial ROS for tumor cell proliferation, survival, and for tumor xenograft growth by stably expressing a hydrogen peroxide (H_2_O_2_) scavenger, peroxiredoxin-5, in the mitochondrial IMS (IMS-Prdx5) in 143B osteosarcoma and HCT116 colorectal cancer cell lines. IMS-Prdx5 attenuates hypoxia-induced ROS signaling as assessed independently in cytosol and IMS, HIF-1α stabilization and activity, and cellular proliferation under normoxic and hypoxic culture conditions. It also suppressed tumor growth *in vivo*. Stable expression of nondegradable HIF-1α only partially rescued proliferation in IMS-Prdx5-expressing cells, indicating that mitochondrial H_2_O_2_ signaling contributes to tumor cell proliferation and survival through HIF-dependent and HIF-independent mechanisms.

Cancer cells generate high levels of oxidative stress as a consequence of oncogene activation, nucleotide biosynthesis, and growth factor receptor signaling (1). Mitochondria contribute to this redox stress by generating reactive oxygen species (ROS) along the electron transport chain (ETC), which releases superoxide to the mitochondrial matrix (arising from complexes I, II, or III) or the intermembrane space (IMS) (from complex III) (2). The superoxide is then dismuted to hydrogen peroxide (H_2_O_2_) by manganese superoxide dismutase (MnSOD) and copper-zinc-superoxide dismutase (CuZnSOD) in those compartments, respectively. Mitochondrial H_2_O_2_ that reaches the cytosol has been reported to participate in signaling that contributes to prosurvival responses such as the activation of hypoxia-inducible factors (HIFs) or NF-κB (3, 4), although other studies dispute this (5, 6). Notably, the combined oxidative stresses that cancer cells endure also render them vulnerable to exogenous redox stresses, such as those induced by chemotherapeutic agents (7, 8).

Redox homeostasis is highly compartmentalized in the cell, with independent systems functioning in the mitochondria, cytosol, endoplasmic reticulum, nucleus, and other subcellular compartments (9). Some studies of cancer cells have used nonspecific antioxidants that can affect multiple subcompartments but these have yielded disparate findings. For example, 1 study using N-acetyl cysteine found that this antioxidant suppressed tumor growth in mice by inhibiting the oxidant signals that contribute to HIF-1α stabilization (10) but others have reported that antioxidants accelerate tumor initiation, growth and metastatic potential (6, 11).

An alternative approach to evaluate the role of mitochondrial ROS in cancer cells is to use ETC inhibitors. These compounds suppress tumor cell growth and survival (12) but they increase ROS generation at sites proximal to the inhibition while decreasing production at distal sites. Moreover, it is difficult to know whether their antitumor effects are caused by their ability to alter mitochondrial ROS generation or to their inhibitory effects on oxidative phosphorylation and ATP synthesis (13). Another approach has been to target antioxidant compounds, such as MitoQ or MitoTEMPO to the mitochondrial matrix by adding a cationic triphenylphosphonium (TPP^+^) group. Some studies have shown that MitoQ is highly toxic to breast cancer cells relative to normal controls (14), whereas others have found that MitoQ and MitoTEMPO have no effect on malignant melanoma or lung cancer progression in mice (5). In either case, questions have been raised about the inhibitory effects of these TPP^+^-containing compounds on mitochondrial respiration (15). Importantly, antioxidants targeted to the mitochondrial matrix presumably cannot act on ROS released from the inner membrane to the IMS.

To understand the role of H_2_O_2_ release from cancer cell mitochondria in terms of its effects on cell signaling, proliferation, and tumor growth, we stably expressed an H_2_O_2_ scavenger—peroxiredoxin-5 (Prdx5)—in the IMS in two human cancer cell lines. This enzyme is normally expressed in the matrix, so its expression in the IMS allows it to scavenge H_2_O_2_ in transit from the inner membrane to the cytosol, without affecting oxidative phosphorylation or oxygen consumption (16).

## Results

### Expression of IMS-Prdx5 in 143B cells alters hypoxia-induced redox signaling in subcellular compartments

We targeted Prdx5 to the mitochondrial IMS-Prdx5 in order to scavenge H_2_O_2_ arising from the ETC, to determine the effect of these signals on HIF activation in tumor cells. Prdxs 5 are thiol-dependent peroxidases that attack the O–O bond of hydrogen peroxide using a peroxidatic cysteine (Cys) at the active site, resulting in the formation of a sulfenic acid that must be reduced by another Cys thiol. Prdx5 is a mammalian atypical 2-Cys peroxiredoxin that contains the resolving thiol within the same protein, allowing it to reduce its own sulfenic acid to a disulfide bond. The disulfide is then reduced by thioredoxins, restoring its catalytic activity (17). Prdx5 was chosen for these studies based on its ability to function as a monomeric H_2_O_2_ scavenger.

**Figure 1:**
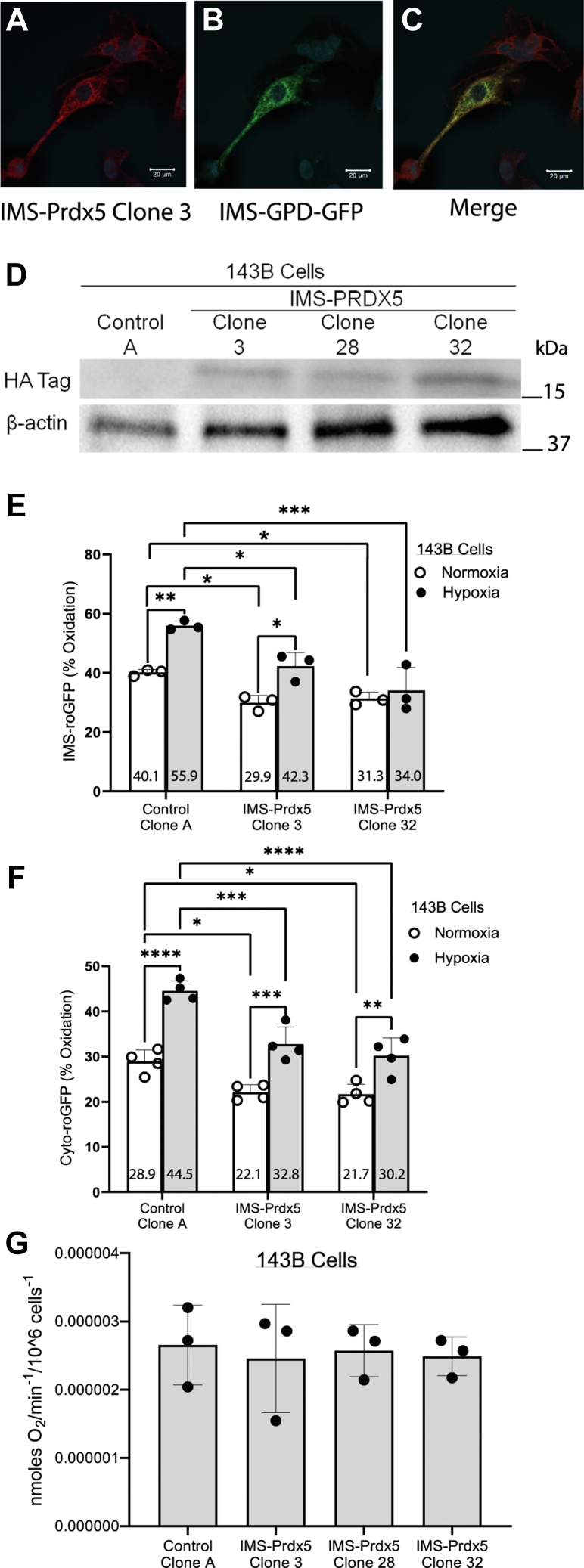
**Stable expression of IMS-Prdx5 in 143B osteosarcoma cells alters normoxic oxidant stress and abrogates hypoxia-induced oxidant changes in multiple cellular compartments.***A*–*C*, confocal images showing immunostaining for the HA Tag on IMS-Prdx5 (*red*), IMS-roGFP fluorescence (*green*) and colocalization (merge) in Clone 70 cells. Glycerol-3-phosphate dehydrogenase (GPD) is embedded in the inner mitochondrial membrane. The IMS-roGFP was tethered to the c terminus of GPD, which extends from the membrane into the IMS. The scale bars represent 20 μm. *D*, representative Western blot of monoclonal cell lines showing differing levels of IMS-Prdx5 expression. *E*, 143B cells were exposed to normoxia or hypoxia for 12 to 30 h prior to assessment of live cell IMS-roGFP oxidation on an epifluorescence microscope (n = 3 for each condition). *F*, cytosolic roGFP oxidation in normoxia or hypoxia (n = 4 for each condition). *G*, oxygen consumption of control Clone A 143B cells and IMS-Prdx5 expressing clonal cells (n = 3 each). Data shown as mean ± SEM. ^‡^*p* ≤ 0.05 *versus* normoxic Control Clone A. +*p* ≤ 0.05 *versus* hypoxic Control Clone A. ∗*p* ≤ 0.05 *versus* respective normoxic value. IMS, intermembrane space; Prdx5, peroxiredoxin-5; roGFP, redox-sensitive GFP.

We targeted Prdx5 to the mitochondrial IMS using a 57-amino acid sequence from the mouse smac/Diablo protein (18). Upon translocation from the cytosol into the IMS the first 53 amino acids of the sequence are cleaved off, leaving four additional amino acids on the N terminus of the Prdx5 protein. We previously confirmed successful expression in the IMS using this targeting sequence by employing immunogold labeling and electron microscopy (16). Clonal expansion of 143B osteosarcoma cell lines stably transfected with either IMS-Prdx5 or the empty expression vector was performed after neomycin selection. IMS localization was then reconfirmed by immunostaining for IMS-Prdx5 and for mitochondrial redox-sensitive GFP (roGFP) tethered to the C terminus of glycerol phosphate dehydrogenase, which resides in the IMS (19). Cells transfected with an empty expression vector failed to express IMS-Prdx5. Cells transfected with the IMS-Prdx5 vector expressed IMS-Prdx5 that colocalized with IMS-roGFP (Fig. 1, *A*–*C*). Immunoblots of monoclonal cell lines revealed differing levels of IMS-Prdx5 expression, with Clone 32 expressing the highest level, followed by Clone 3 and Clone 28 (Fig. 1*D*).

Subcellular compartmental oxidant signaling was evaluated in monoclonal lines of 143B cells exposed to prolonged hypoxia (12–30 h at 1.5% O_2_) or normoxia (21% O_2_) using roGFP, a ratiometric, thiol redox-sensitive protein sensor targeted to the IMS (IMS-roGFP) (Fig. 1*E*). In Control Clone A, hypoxia caused an increase in IMS oxidant signaling compared with normoxic baseline levels. This finding is consistent with previous studies showing hypoxia-induced increases in IMS oxidant stress in primary cells (20). Basal oxidation levels in IMS-Prdx5 Clones 3 and 32 were lower under normoxic and hypoxic conditions than Control Clone A. The hypoxia-induced increase in oxidation in the IMS was attenuated in Clone 32, which expressed the highest levels of IMS-Prdx5.

If ROS released to the IMS from complex III affect oxidant signaling in the cytosol during hypoxia, then IMS-Prdx5 should attenuate that response. To test this, 143B cells were exposed to hypoxia or normoxia and their cytosolic oxidation levels were assessed using roGFP expressed in that compartment. Control Clone A exhibited an increase in oxidation during hypoxia (Fig. 1*F*). This result is consistent with previous studies using redox-sensitive sensors in 143B cells subjected to hypoxia (21). Compared with control cells, Clones 3 and 32 displayed a significant attenuation of cytosolic oxidant signaling both under normoxic and hypoxic conditions. However, both IMS-Prdx5 clones still exhibited increases in hypoxia-induced oxidant stress compared with their respective normoxic baselines.

Oxygen consumption measurements revealed that IMS-Prdx5 expression does not alter cellular respiration rates in any of the 143B clones (Fig. 1*G*).

**Supporting Figure S1:**
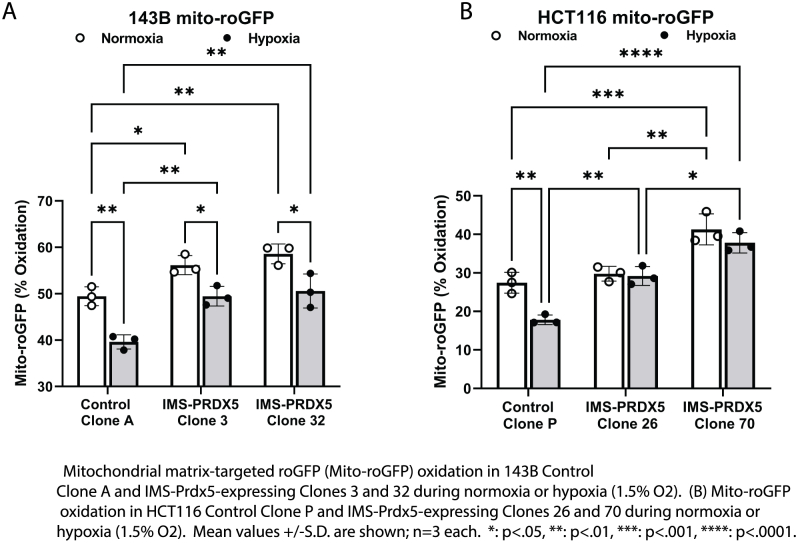


In additional studies, the effect of IMS-Prdx5 on mitochondrial matrix oxidant status was assessed using a matrix-targeted roGFP (mito-roGFP) sensor in 143B cells. Compared with normoxia, hypoxic cells exhibited a lower level of mito-roGFP oxidation (Fig. S1*A*).

### IMS-Prdx5 expression in 143B cells attenuates HIF-1α stabilization and activity in a dose-dependent manner

**Figure 2:**
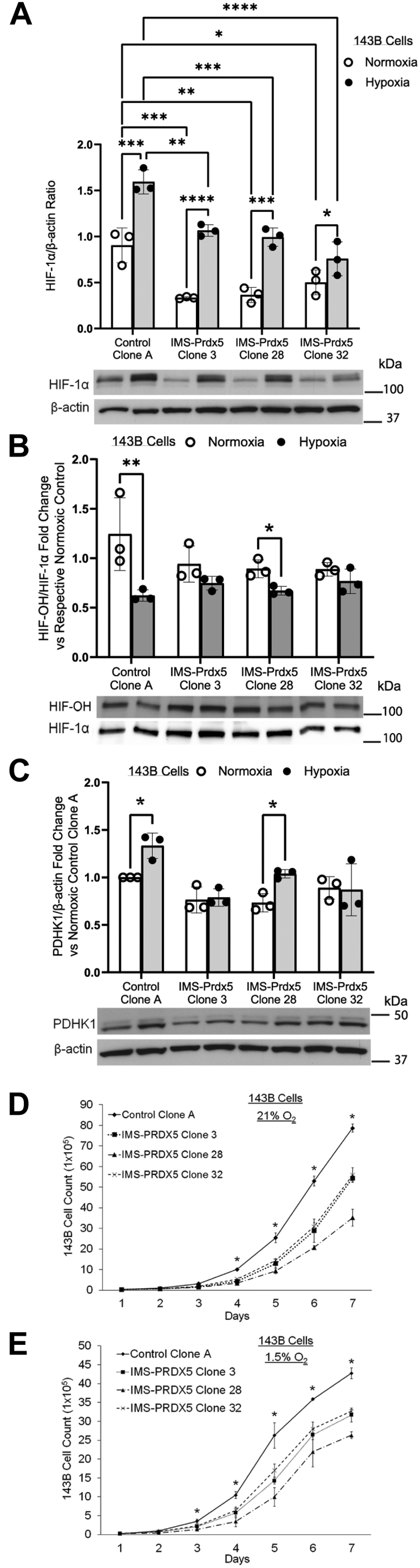
**Stable IMS-Prdx5 expression in 143B osteosarcoma cells attenuates normoxic and hypoxic HIF-1α stabilization and activity, as well as the hypoxia-induced decrease in hydroxylated HIF-1α, in a dose-dependent manner.***A*, cells were exposed to hypoxia for 8 h prior to collecting lysates for detection of HIF-1α by Western blot. ^‡^*p* ≤ 0.05 *versus* normoxic Control Clone A. +*p* ≤ 0.05 *versus* hypoxic Control Clone A. ∗*p* ≤ 0.05 *versus* respective normoxic value. *B*, cells were exposed to hypoxia for 4 h in the presence of 10 μM MG132, a proteasome inhibitor, prior to collecting lysates for detection of hydroxylated HIF-1α. Expression levels of HIF-1α-OH relative to total HIF-1α in the same blot are shown. The super shifted bands in the HIF-1α-OH blot represent glycosylated HIF-1α. ∗*p* ≤ 0.05 *versus* respective normoxic control. *C*, cells were exposed to hypoxia for 8 h prior to collecting lysates for PDHK1 detection by Western blot. ∗*p* ≤ 0.05 *versus* respective normoxic control. 2 × 10^4^ cells were seeded in parallel in 10 cm dishes and one dish was counted each day for 7 days in (*D*) 21% O_2_ or (*E*) 1.5% O_2_. ∗ *p* ≤ 0.05 for Control Clone A *versus* all IMS-Prdx5 clones. Data shown as mean ± SEM (n = 3 for all groups). HIF, hypoxia-inducible factor, IMS, intermembrane space; PDHK1, pyruvate dehydrogenase kinase-1; Prdx5, peroxiredoxin-5.

To determine whether H_2_O_2_ scavenging in the IMS attenuates HIF-1α stabilization and activity, 143B cells were exposed to hypoxia or normoxia for 8 h. Cell lysates were collected and Western blots were performed to measure relative HIF-1α levels. IMS-Prdx5 Clones 3, 28, and 32 all exhibited lower normoxic and hypoxia-induced levels of HIF-1α than Control Clone A (Fig. 2*A*). Clone 32, which expressed the highest levels of IMS-Prdx5, exhibited the smallest hypoxia-induced increase in HIF-1α compared to other clones.

To determine whether hypoxia-induced ROS affect HIF-1α stabilization by altering HIF prolyl hydroxylase (PHD) function, we assessed levels of hydroxylated HIF-1α (HIF-OH) at Pro^564^ *via* Western blot. As expected, hypoxia decreased HIF-OH in Control Clone A (Fig. 2*B*). IMS-Prdx5 Clone 28, which expressed a low level of the antioxidant, was unable to affect this hypoxia-induced decrease in HIF-OH. However, higher IMS-Prdx5-expressing Clones 3 and 32 attenuated this decrease in HIF hydroxylation, suggesting that hypoxia-induced mitochondrial H_2_O_2_ acts upstream of the PHDs in the hypoxic HIF signaling pathway.

Having shown that IMS-Prdx5 expression attenuates HIF-1α stabilization, we next sought to determine if HIF-1α activity was also attenuated. Using pyruvate dehydrogenase kinase-1 (PDHK1), a well-defined HIF-1 target gene that is frequently involved in cancer metabolism reprogramming (22, 23), we found that Control Clone A PDHK1 is upregulated after 8 h of hypoxic exposure (Fig. 2*C*). Again, IMS-Prdx5 Clone 28 was unable to blunt this response but Clones 3 and 32 attenuated the hypoxia-induced HIF-1α-regulated increase in PDHK1 expression. The effects of IMS-Prdx5 on cell proliferation were also assayed under normoxic (21% O_2_) and hypoxic (1.5% O_2_) conditions (Fig. 2, *D* and *E*, respectively). In normoxia, Control Clone A exhibited a proliferative advantage over all IMS-Prdx5 clones after 4 days of growth. This growth advantage was further enhanced under hypoxic conditions, where control cells propagated significantly faster than IMS-Prdx5 clone cells after only 3 days of growth.

### Effects of IMS-Prdx5 expression are recapitulated in HCT116 colon cancer cells

**Figure 3:**
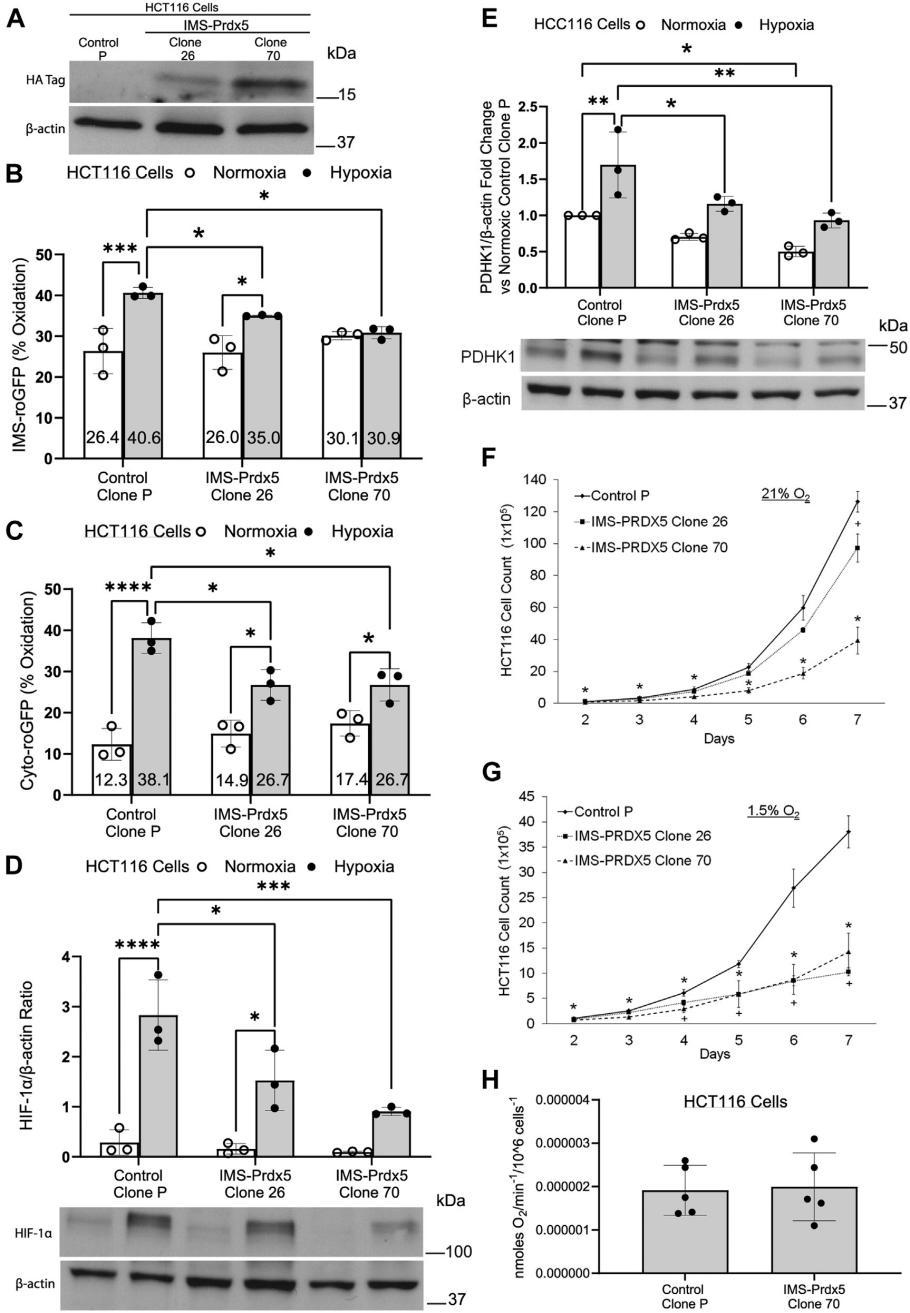
**IMS-Prdx5 expression attenuates hypoxic responses in HCT116 colon cancer cells.***A*, representative Western blot showing levels of IMS-Prdx5 in monoclonal cell lines. *B*, IMS and (*C*) cytosolic roGFP studies in normoxia and hypoxia. *D*, HIF-1α Western blot data after 8 h of hypoxia. *E*, PDHK1 Western blot data after 12 h of hypoxia. *F*, cell growth in tissue culture in 21% O_2_ or (*G*) 1.5% O_2_, or hypoxia. In each case, 2 × 10^4^ cells were seeded in parallel in 10 cm dishes and one dish was counted each day for 7 days. For (*A*–*E*): ^‡^*p* ≤ 0.05 *versus* normoxic Control Clone P, +*p* ≤ 0.05 *versus* hypoxic Control Clone P, ∗*p* ≤ 0.05 *versus* respective normoxic value. For (*F* and *G*): ∗*p* ≤ 0.05 for IMS-Prdx5 Clone 70 *versus* Control Clone P, +*p* ≤ 0.05 for IMS-Prdx5 Clone 26 *versus* Control Clone P. Data shown as mean ± SEM (n = 3 for all experiments). *H*, cellular oxygen consumption measurements by HCT116 Control P cells and IMS-Prdx5 Clone 70 cells suspended in media (n = 5 each; *p* = N.S.). HIF, hypoxia-inducible factor; IMS, intermembrane space; PDHK1, pyruvate dehydrogenase kinase-1; Prdx5, peroxiredoxin-5; roGFP, redox-sensitive GFP.

To ensure that the previous results were not unique to 143B cells we tested the effect of IMS-Prdx5 expression in HCT116 colorectal cancer cells, whose cellular proliferation and tumorigenicity is largely driven by HIF-1 (24). Again, varying expression levels of IMS-Prdx5 were obtained among clones, with Clone 70 expressing higher levels than Clone 26. Using quantitative RT-PCR measurements in HCT116 clone 70 cells, the expression of the IMS-Prdx5 was found to be 47% of the native Prdx5 expression in the cells. A comparison of the Ct values (Prdx5 *versus* IMS-Prdx5) yielded a *p* value of 0.0168. Subcellular levels of oxidation were again assessed using the IMS-roGFP sensor. The IMS-Prdx5 clones attenuated the hypoxia-induced increase in oxidant levels in a dose-dependent manner, with Clone 70 completely abolishing the increase (Fig. 3*B*). Both clones also significantly mitigated the hypoxia-induced levels of cytosolic-roGFP oxidation, though this still increased relative to their respective normoxic baselines (Fig. 3*C*). As seen with the 143B cell clones, mito-roGFP oxidation was lower in hypoxic HCT116 cell clones than normoxia (Fig. S1*B*).

When compared to Control Clone P cells, Clones 26 and 70 attenuated hypoxia-induced stabilization of HIF-1α in a dose-dependent manner (Fig. 3*D*). We again assessed HIF-1 activity by measuring the protein expression of PDHK1 (a target gene of HIF-1) and observed that the IMS-Prdx5 clones displayed significantly less PDHK1 expression in hypoxia than Control P cells, with Clone 70 also exhibiting significantly less PDHK1 under normoxic conditions (Fig. 3*E*). Quantitative RT-PCR measurements of PDHK1 mRNA expression in Control P cells show an increased expression under hypoxic conditions *versus* normoxia (Fig. S2*A*) as well as a decrease in PDHK1 mRNA expression in clone 70 cells under hypoxia when compared to Control P cells (Fig. S2*B*). Cell proliferation assays again revealed a growth disadvantage of IMS-Prdx5 clones *versus* Control P cells in both normoxia and hypoxia (Fig. 3, *F* and *G*, respectively). Cellular oxygen consumptions were not different between Control P cells and IMS-Prdx5 Clone 70 cells (Fig. 3*H*). Control P exhibited a proliferative advantage over Clone 70 at every time point studied in both normoxia and hypoxia. Control P cells also showed a significant growth advantage over Clone 26 in normoxia after 7 days of propagation but that difference was enhanced in hypoxia such that significant differences were apparent after only 4 days. These data show that the effects of IMS-Prdx5 expression seen in 143B cells are sustained in a cell line whose cellular proliferation is positively regulated by HIF-1α signaling.

### Attenuation of hypoxia-induced ROS signaling and HIF-1α stabilization by IMS-Prdx5 requires its catalytic activity

**Figure 4:**
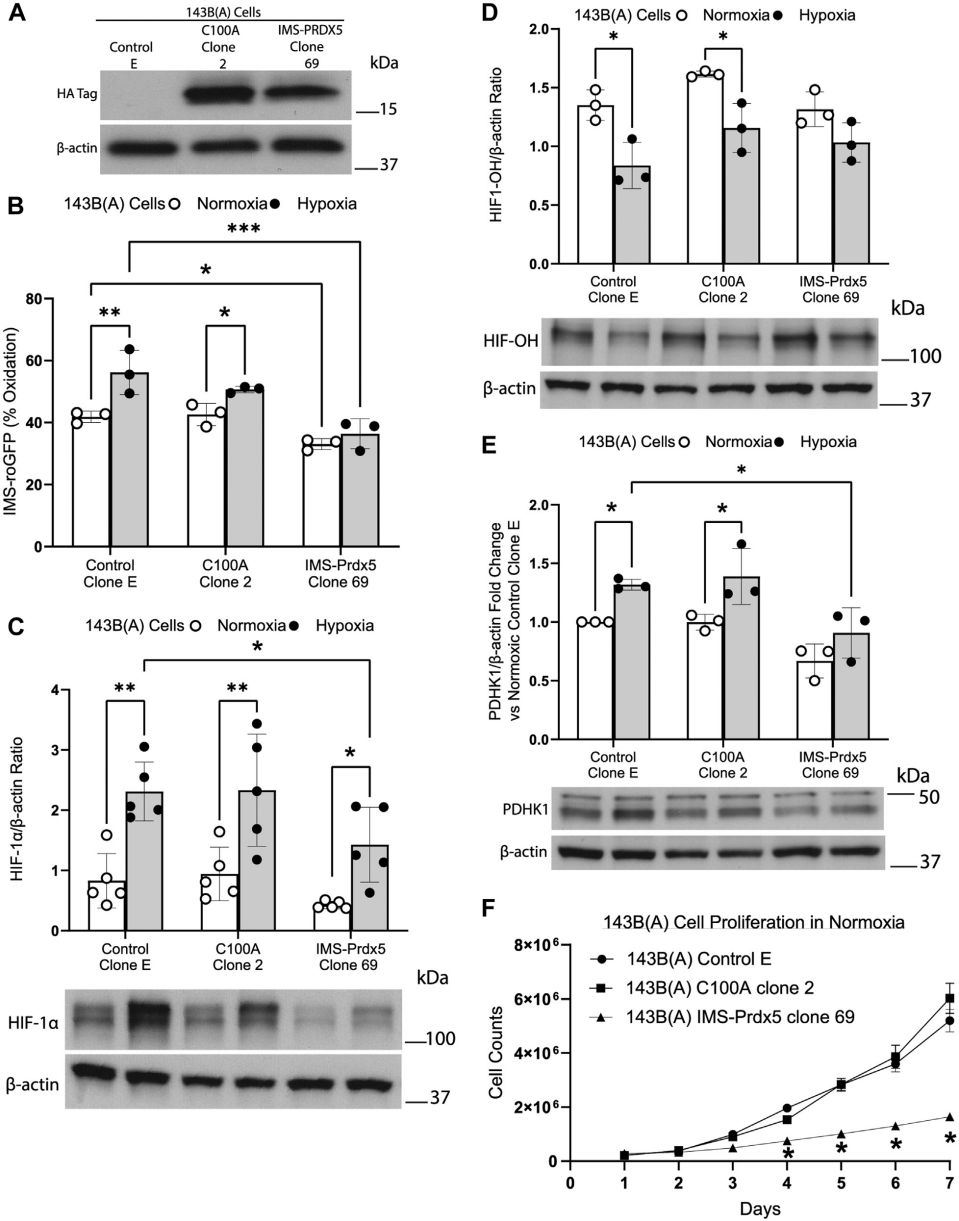
**Attenuation of hypoxia-induced ROS signaling and HIF-1α stabilization and activity by IMS-Prdx5 is dependent on its intact enzymatic activity.***A*, representative Western blot showing levels of IMS-Prdx5 or C100A expression. *B*, IMS roGFP after 12 to 30 h hypoxic exposure. *C*, HIF-1α Western blot data after 8 h of hypoxia. *D*, HIF-OH Western blot data, cells were exposed to 4 h of hypoxia in the presence of 10 μM MG132 a proteasome inhibitor, prior to collecting lysates for detection of hydroxylated HIF-1α. *E*, PDHK1 Western blot data after 12 h of hypoxia. *F*, 1.0 × 10^5^ cells were seeded in parallel in 10 cm dishes and one dish was counted each day for 7 days. Cell growth curves of 143B(A) control E cells, 143B(A) C100A clone 2 cells, or 143B(A) IMS-Prdx5 clone 69 cells under normoxic conditions. For (*B*, *C* and *E*): ^‡^*p* ≤ 0.05 *versus* normoxic Control Clone E. +*p* ≤ 0.05 *versus* hypoxic Control Clone E. ∗*p* ≤ 0.05 *versus* respective normoxic value. For (*D*) ∗*p* ≤ 0.05 *versus* respective normoxic control. For (*F*) ∗*p* ≤ 0.01 *versus* respective normoxic143B(A) control E cells on day 4 and *p* ≤ 0.0001 on days 5 to 7. Data shown (*B*–*E*) as mean ± SEM (n = 3 for all experiments). Data shown for (*F*) as mean ± SEM (n = 5 for the experiment). IMS, intermembrane space; Prdx5, peroxiredoxin-5; roGFP, redox-sensitive GFP; ROS, reactive oxygen species.

We then tested whether the H_2_O_2_ scavenging activity of IMS-Prdx5 was required for the attenuation of HIF-1α stabilization during hypoxia. An enzymatically inactive version of the IMS-Prdx5 antioxidant was generated by mutating the active-site cysteine to an alanine (C100A). Note that the PRDX5 mutant used, C100A, corresponds to mutagenesis of the PRDX5 peroxidatic cys (Cys47) since it includes the first 1-53 aa mitochondrial localization signaling sequence. The 143B Control A cells used in Figures 1 and 2 were then transfected with either an empty expression vector, a vector with the C100A mutation or with the catalytically active IMS-Prdx5, and clones were selected using zeocin (referred to as 143B(A) cells). Figure 4*A* shows protein expression levels of various clones. Studies of redox signaling using IMS-roGFP confirmed that C100A Clone 2 exhibited comparable normoxic and hypoxia-induced oxidation levels compared to Control E cells. By contrast, IMS-Prdx5 Clone 69 showed significantly decreased levels of normoxia- and hypoxia-induced oxidation and a complete loss of hypoxia-induced increase in IMS-roGFP oxidation (Fig. 4*B*). Control E and C100A Clone 2 exhibited comparable levels of normoxia- and hypoxia-induced HIF-1α and PDHK1 expression, whereas in IMS-Prdx5-expressing Clone 69 the expression levels were significantly lower (Fig. 4, *C* and *E*). Furthermore, C100A Clone 2 exhibited similar hypoxia-induced decreases in HIF-OH compared with Control E, while that decrease was attenuated in IMS-Prdx5 Clone 69 (Fig. 4*D*). These experiments confirm that the inhibitory effects of IMS-Prdx5 on hypoxia-induced ROS signaling and HIF-1α stabilization and activity are dependent on the H_2_O_2_ scavenging function of the enzyme.

### IMS-Prdx5 attenuates tumor growth *in vivo*

**Figure 5:**
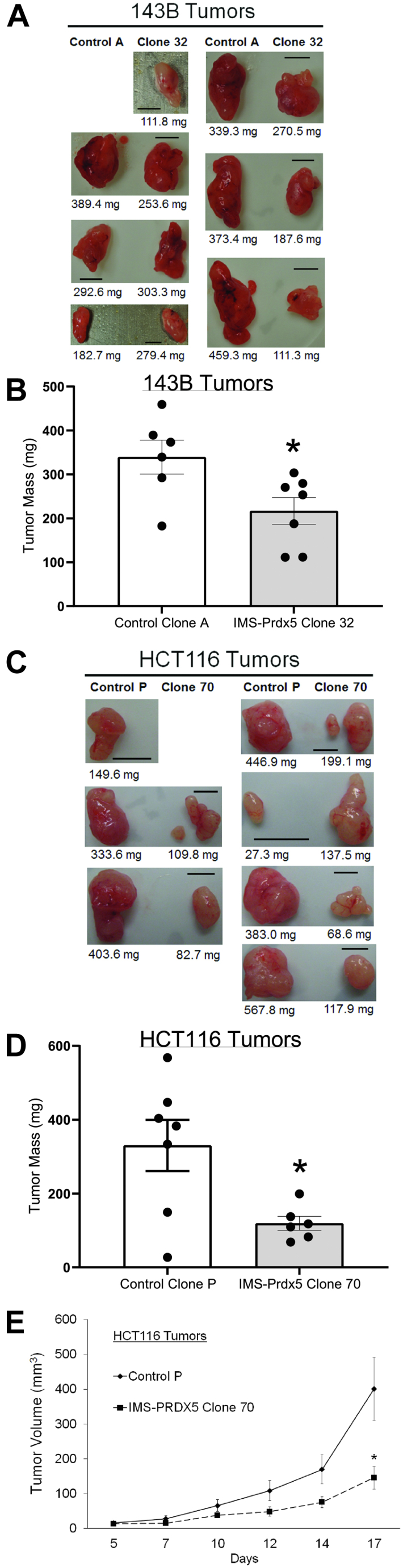
**Stable expression of IMS-Prdx5 attenuates tumor growth *in vivo*.***A*, pictures of 143B Control A and IMS-Prdx5 Clone 32 tumors. Paired images illustrate relative tumor sizes from contralateral sides of the same mouse. One mouse failed to develop a Control A tumor, so no image is provided (n = 6–7 for each group). The scale bar represents 5 mm. *B*, average mass of 143B tumors. *C*, pictures of HCT116 Control P and IMS-Prdx5 Clone 70 tumors. Paired images illustrate relative tumor sizes from contralateral sides of the same mouse (n = 6–7 for each group). The scale bar represents 5 mm. One mouse failed to develop a Clone 70 tumor, so no image is provided. *D*, average mass of HCT116 tumors. *E*, average volume of HCT116 tumors. Data shown as mean ± SEM. ∗*p* ≤ 0.05 *versus* control value. IMS, intermembrane space; Prdx5, peroxiredoxin-5.

After finding that IMS-Prdx5 expression attenuates hypoxia-induced ROS signaling and HIF-1 activation *in vitro*, we sought to test whether similar suppression of growth could be seen *in vivo*. 143B Control A and IMS-Prdx5 Clone 32 cells were injected subcutaneously into the flanks of nude mice. This xenograft model was selected because tumor growth is highly dependent on HIF-dependent vascular endothelial growth factor expression, providing a sensitive test of the hypoxia–sensing pathway (25). Tumors were monitored for 2 to 3 weeks and then harvested (Fig. 5*A*). The mass of tumors expressing the IMS-Prdx5 was significantly smaller than in the control cell tumors (Fig. 5*B*). HCT116 Control P cells and IMS-Prdx5 Clone 70 cells were injected in a similar fashion (Fig. 5*C*). IMS-Prdx5 tumors were smaller and their growth rates were slower than for control cells (Fig. 5, *D* and *E*). These findings show that IMS-Prdx5 expression affects cellular proliferation *in vivo*, using the subcutaneous xenograft model known to produce severe hypoxia during early tumor growth.

### IMS-Prdx5 decreases *in vivo* tumor HIF-1α protein levels and activity

**Figure 6:**
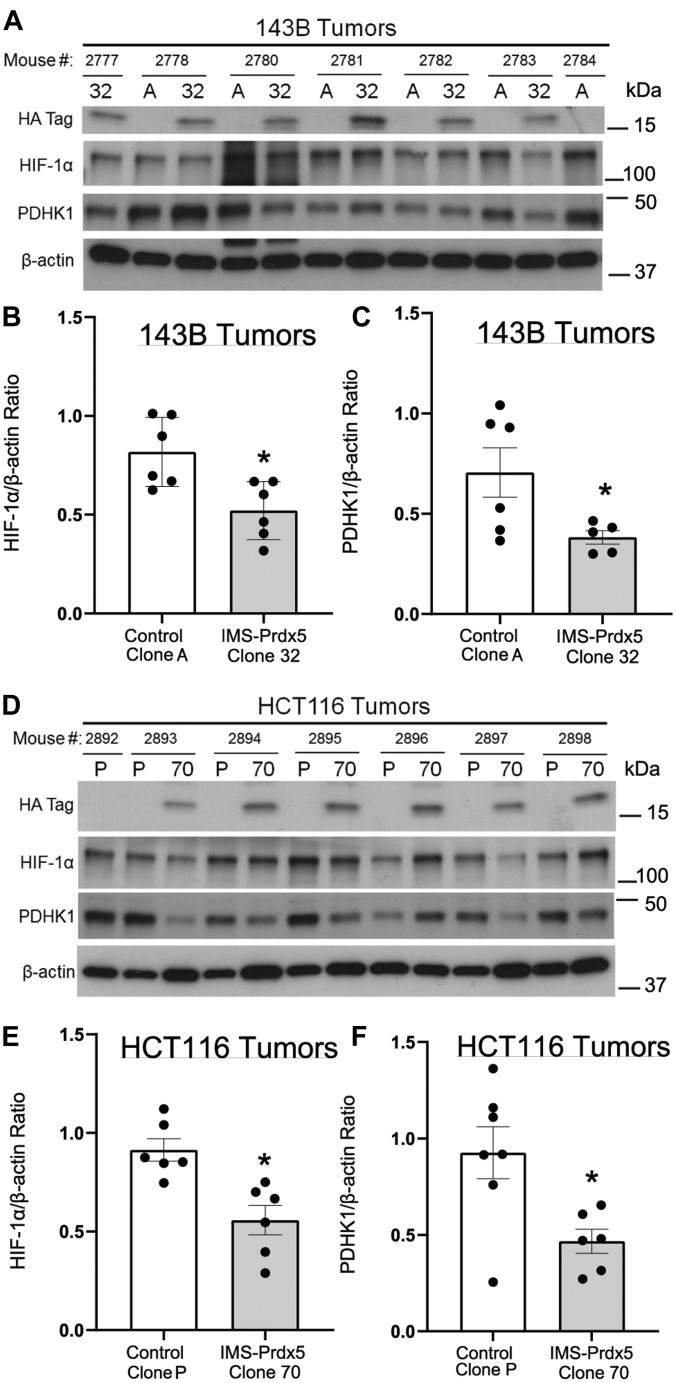
**Expression of IMS-Prdx5 significantly decreases *in vivo* tumor protein levels of HIF-1α and a marker of HIF-1α activity.***A*, Western blots of protein lysates from 143B tumors. No Control A tumor grew in mouse 2777, so only the Clone 32 tumor sample is shown. *B*, graphical summary of amounts of HIF-1α and (*C*) PDHK1, normalized to β-actin (n = 5–6 in each group). No Clone 70 tumor grew in mouse 2892, so only the Control P sample is shown. *D*, Western blots of protein lysates from HCT116 tumors. *E*, graphical summary of amounts of HIF-1α and (*F*) PDHK1, normalized to β-actin (n = 6 in each group). Data shown as mean ± SEM. ∗*p* ≤ 0.05 *versus* control value. HIF, hypoxia-inducible factor; IMS, intermembrane space; PDHK1, pyruvate dehydrogenase kinase-1; Prdx5, peroxiredoxin-5.

Tumors were analyzed to determine the effects of IMS-Prdx5 expression on the HIF signaling pathway (Fig. 5). Immunoblotting of protein lysates from the 143B tumors using an antibody against the hemagglutinin (HA) tag confirmed that the IMS-Prdx5 tumors continued to express the IMS-Prdx5 protein *in vivo* (Fig. 6*A*). These tumors also exhibited significantly decreased levels of HIF-1α and PDHK1 protein, indicating that IMS-Prdx5 suppresses HIF-1α stabilization and activity (Fig. 6, *B* and *C*). We performed qRT-PCR for PDHK1 in 143B tumors (Fig. S2*C*). Tumor PDHK1 expression tended to be smaller in the IMS-Prdx5 Clone 32 tumors than in Control A cells, but this was not statistically significant, possibly because of an increase in PDHK1 in tumor stromal cells, which would not have contained the IMS-Prdx5 protein and would still be responsive to the hypoxic tumor microenvironment. Immunoblots depicting expression of IMS-Prdx5 in the HCT116 tumor lysates (Fig. 6*D*) similarly demonstrated that the antioxidant enzyme significantly decreases HIF-1α stabilization (Fig. 6*E*) and transcriptional activity (Fig. 6*F*). These data indicate that IMS-Prdx5 acts as a tumor suppressor by inhibiting mitochondrial H_2_O_2_ signaling (Fig. 6*G*).

### Scavenging of mitochondrial H_2_O_2_ signaling decreases cellular proliferation

**Figure 7:**
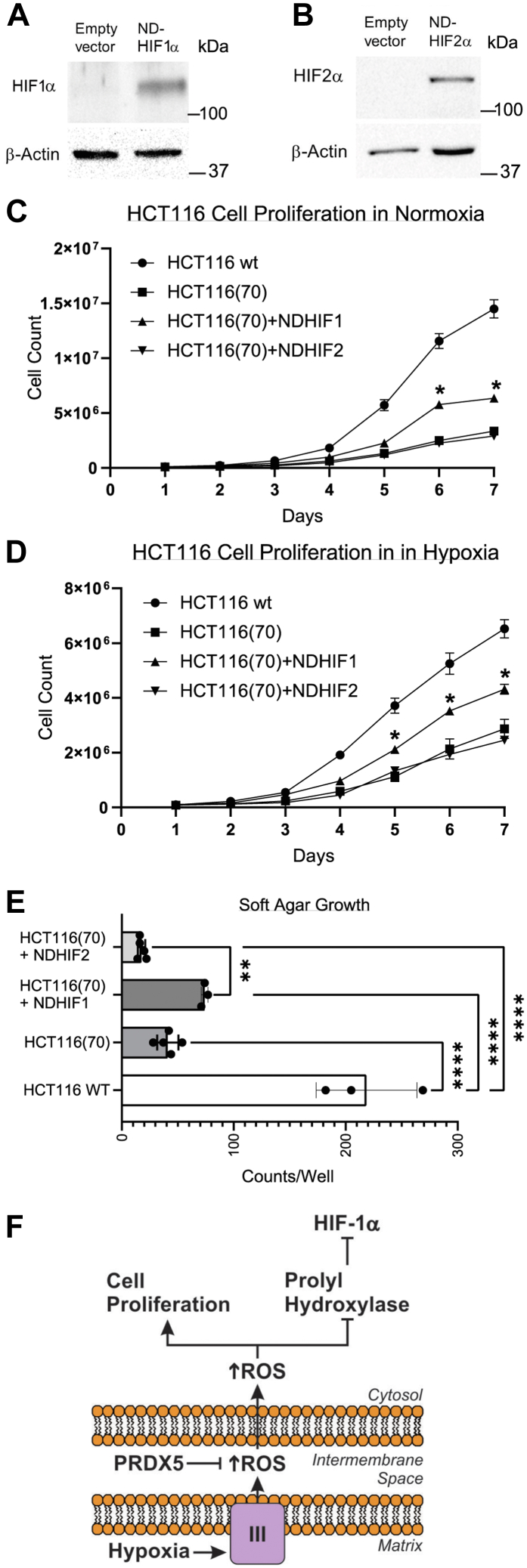
**Scavenging mitochondrial oxidant signals decreases cellular proliferation in a manner partially rescued by stable expression of HIF-1α.***A*, normoxic protein expression of HIF-1α in HCT116 (Clone 70) cells stably transfected with the empty vector or the ND-HIF-1α. *B*, normoxic HIF-2α expression in HCT116 (Clone 70) cells stably transfected with the empty vector or the ND-HIF-2α. 1.0 × 10^5^ cells were seeded in parallel in 10 cm dishes and one dish was counted each day for 7 days in (*C*) and (*D*). *C*, cell growth curves of WT HCT116 cells or HCT116 (Clone 70) cells or Clone 70 cells stably expressing ND-HIF-1α or ND-HIF-2α under normoxic conditions (n = 6 each, mean values ± SEM). *D*, cell growth curves of WT HCT116 cells or HCT116 (Clone 70) cells or Clone 70 cells stably expressing ND-HIF-1α or ND-HIF-2α under hypoxic (1.5% O_2_) conditions (n = 6 each, mean values ± SEM). *E*, soft agar colony formation of HCT116 Clone 70 cells transfected with a nondegradable form of HIF-1α, HIF-2α, or an empty vector, compared to WT HCT116 cells under normoxia (n = 3–5, mean values ± SEM) ∗∗*p* < 0.01, ∗∗∗∗*p* < 0.0001. *F*, model of decreased tumorigenesis by IMS-Prdx5 *via* ROS scavenging and attenuation of HIF-1 signaling. HIF, hypoxia-inducible factor; IMS, intermembrane space; Prdx5, peroxiredoxin-5; ROS, reactive oxygen species.

HIF-1 has been reported to drive proliferation in HCT116 cells (24). To test whether the IMS-Prdx5-induced decrease in cellular proliferation was caused by the observed decrease in HIF-1α stabilization, we restored HIF-1 activity by stably transfecting HCT116 Clone 70 cells with a construct in which Pro^402^ and Pro^564^ of HIF-1α were mutated, rendering the protein insensitive to modification by HIF PHD (26). Like HIF-1α, HIF-2α is negatively regulated by PHD and its stabilization in hypoxia would also be suppressed by the IMS-Prdx5 construct. To detect a possible role of decreased HIF-2 signaling in the attenuated cell growth, we also stably transfected Clone 70 cells with a nondegradable form of HIF-2α. Stable transfection resulted in an increase in nondegradable HIF-1α (Fig. 7*A*) and HIF-2α (Fig. 7*B*) during normoxia. Compared with WT HCT116 cells, cell growth of HCT116 Clone 70 cells was significantly inhibited under normoxic conditions (Fig. 7*C*). Stable expression of nondegradable HIF-1α in the Clone 70 cells significantly increased growth at 6 and 7 days compared with Clone 70 cells but growth remained suppressed compared with the WT cells. Growth of Clone 70 cells expressing nondegradable HIF-2α was undistinguishable from that of Clone 70 cells, under normoxic conditions. Similar trends were observed when the cells were maintained at 1.5% O_2_, where expression of nondegradable HIF-1α increased growth compared with Clone 70 cells but did not restore growth to that of the WT cells. Again, nondegradable HIF-2α expression did not affect growth rates of the Clone 70 cells. Finally, colony formation during growth in soft agar was assessed using the same cell lines. Clone 70 cells grew fewer colonies than WT cells. Although expression of nondegradable HIF-1α tended to increase colony formation, this did not reach statistical significance. However, Clone 70 cells expressing nondegradable HIF1α formed more colonies than Clone 70 cells with stable expression of HIF-2α. These findings indicate that loss of HIF-1α activity in the cells expressing IMS-Prdx5 was responsible for some, but not all of, the suppression of cell growth. Thus, it appears that mitochondrial H_2_O_2_ signals control cellular proliferation through both HIF-1-dependent and HIF-1-independent mechanisms.

## Discussion

Tumor cells endure increased levels of oxidant stress as a consequence of oncogene activation as well as from growth factor receptor signaling (1, 27). Simultaneously their antioxidant defenses are undermined by competing demands for NADPH, which is needed both for antioxidant defenses and for the biosynthesis of deoxyribonucleotides by ribonucleoside reductase (28, 29). Thus, tumor cells must negotiate a careful balance between too much oxidant stress, which can damage DNA, lipids, and proteins, and too little, which may hinder redox signals required for growth factor receptor signaling (8, 30). The role of ROS in cancer cells has also been controversial, with some studies reporting that oxidant signaling promotes tumor growth (10), while others suggesting the opposite (6, 11) and yet others showing no effect (5).

Mitochondria have been reported to contribute to cancer progression through the generation of redox signals (1, 31). Mitochondrial ROS arise primarily from the ETC, which can release superoxide to the matrix compartment or the IMS. A key role for mitochondrial ROS signaling occurs during hypoxia, which stimulates the release of ROS from complex III (3, 32, 33). These signals promote the stabilization of HIF-1α; subsequent HIF transcriptional activity leads to the expression of multiple genes that contribute to cancer cell survival, altered metabolism, migration, and metastatic phenotype (34, 35). Accordingly, loss of complex III function during hypoxia leads to decreases in ROS signaling, decreases in hypoxic HIF-1α stabilization (3, 21, 36) and decreases in tumor cell growth *in vitro* and *in vivo* (37).

However, current understanding of the importance of mitochondrial ROS for tumor cell biology is based on experiments that employ imperfect methodologies. Some studies have used ρ^0^ tumor cell lines lacking a functional ETC; these cells fail to generate ROS signals or to stabilize HIF-1α during hypoxia (3). Other studies have used selective deletion of ETC subunits such as those in complex III and found similar repression of hypoxic ROS signaling, HIF activation (21, 38, 39) and tumor cell growth (37). However, loss of ETC function also inhibits oxidative phosphorylation and ATP generation, so bioenergetic deficiencies in these cells could conceivably explain the decrease in proliferation. Other studies have utilized mitochondria-targeted antioxidant compounds, which localize to the matrix through the addition of a TPP^+^ group. For example, the compound MitoQ was shown to suppress hypoxic HIF-1α stabilization (40), while similarly targeted antioxidants suppressed tumor cell anchorage-independent cell growth (12). However, antioxidants in the matrix might not affect ROS in the IMS, a compartment that is presumably important for redox signaling in the cytosol. Finally, concerns about possible inhibitory effects of TPP^+^ on mitochondrial respiration have been raised (15).

To avoid these issues, we targeted Prdx5, a hydrogen peroxide scavenger, to the IMS of tumor cell mitochondria to determine the significance of mitochondrial ROS signaling for cancer cell proliferation, survival, and *in vivo* tumor growth. As expected, this construct significantly attenuated ROS signaling in both the IMS and the cytosol during hypoxia, in two independent tumor cell lines. The data underscore the importance of mitochondrial oxidant signals for tumor cell proliferation and survival, as the IMS-Prdx5 clones proliferated more slowly than the control cells. Surprisingly, the IMS-Prdx5-expressing cells grew more slowly under both normoxic and hypoxic conditions, suggesting that ROS signals from the mitochondria contribute to cancer cell proliferation through both HIF-dependent as well as HIF-independent pathways. This conclusion is supported by our observation that stable expression of a nondegradable HIF-1α mutant only partially rescued cell proliferation in the IMS-Prdx5 clones, whereas a nondegradable HIF-2α had no effect. Had either of these constructs restored proliferation to the level seen in the WT cell lines, it would have indicated that the loss of HIF signaling was fully responsible for the slowed proliferation. Thus, we conclude that transition of H_2_O_2_ signals through the IMS affects proliferation in tumor cells through HIF-1-dependent and HIF-independent mechanisms that are independent of cellular oxygen levels. While the present study did not identify the HIF-independent mechanisms responsible for these effects, it is possible that mitochondrial H_2_O_2_ signals contribute to cancer cell growth by augmenting redox-dependent growth factor signaling, both in normoxia and in hypoxia.

Our results indicate that only some of the growth suppression in the IMS-Prdx5 cells can be explained by loss of HIF signaling. What factors, other than HIF, could explain this growth suppression? Conceivably, expression of IMS-Prdx5 might interfere with protein folding in the IMS, which involves an oxidant-dependent process. Alternatively, IMS-Prdx5 might interfere with an IMS or cytosolic redox relay that is involved in the regulation of growth but is unrelated to HIF activation. In either case, our results are consistent with prior reports that ROS signaling from the mitochondria is required for cellular oxygen sensing and the HIF response to hypoxia in both primary and cancer cells (3, 9, 20, 21, 32, 38, 41, 42). HIF-1α stabilization, HIF-1 transcriptional activity, and cell proliferation were assessed *in vitro* and *in vivo*, utilizing two different tumor cell lines. The results of these studies are consistent with the model shown in Figure 7*F*. During hypoxia, increased release of superoxide to the IMS leads to the production of H_2_O_2_ in that compartment, which can diffuse to the cytosol to inhibit PHD and enhance HIF-1α stabilization. Scavenging of H_2_O_2_ by IMS-Prdx5 attenuates hypoxia-induced protein thiol oxidation in that compartment, as well as in the cytosol. Loss of this oxidant signal prevents the inhibition of PHD, resulting in the attenuation of hypoxia-induced HIF-1α stabilization, along with HIF-dependent cellular responses. While the current study focused on the role of hypoxia-induced mitochondrial H_2_O_2_ production in the context of tumor cell growth, we believe that the same mechanism underlies the transcriptional responses to hypoxia in normal calls.

Importantly, expression of IMS-Prdx5 attenuated hypoxia-induced oxidant signaling and subsequent HIF responses without affecting electron transport or cellular oxygen consumption rates. Our results extend the work of Orr *et al.* (43), who identified chemical inhibitors of complex III that suppress ROS generation, redox signaling and HIF-1α stabilization without affecting cellular oxygen consumption. Moreover, the effects of IMS-Prdx5 on the cellular responses to hypoxia were dependent on its antioxidant activity, as expression of a mutant lacking the catalytic cysteine residue failed to mimic the inhibitory effects of IMS-Prdx5.

How do mitochondrial oxidant signals regulate HIF hydroxylation? Masson *et al.* (44) studied the effects of peroxide treatment on recombinant HIF prolyl hydroxylase-2 (PHD2) *in vitro*. They reported that PHD2 is not regulated by redox, based on its failure to undergo oxidation in response to H_2_O_2_. However, oxidant signaling frequently involves multiple components in a redox relay system (45). For example, inactivation of STAT3 by oxidants requires peroxiredoxin-2 as a redox intermediary (46). This allows low concentrations of peroxide to oxidize a sensitive target (peroxiredoxin-2), which in turn oxidizes a selective target (STAT3) that is relatively insensitive to direct attack by H_2_O_2_. Further evidence of oxidative regulation of PHD2 comes from Briggs and colleagues, who found that cysteine depletion (induced by paracrine secretion of glutamate) in breast cancer cells led to oxidative inactivation of PHD and stabilization of HIF-1α during normoxia (47). Finally, Lee *et al.* (48) found that oxidants cause dimerization of PHD leading to its inactivation, thereby stabilizing HIF-1α in normoxia. Collectively, these findings support the idea that oxidant signals contribute to the stabilization of HIF-1α and the activation of HIF-dependent transcription. Our data indicate that ROS were acting to inhibit PHD2, as evidenced by the finding that IMS-Prdx5 decreased hypoxia-induced suppression of Pro^564^ hydroxylation. If ROS had no role in the regulation of HIF-1α stabilization, then scavenging ROS in the IMS should not have affected HIF hydroxylation. Based on the work of Masson *et al.* (44), it seems unlikely that H_2_O_2_ signals were acting directly on PHD2 but instead were transmitted through a redox relay intermediary. However, the identity of that intermediary has not been reported.

Some limitations of this study should be noted. First, we used transfection and growth in selection media, followed by screening, to identify 143B and HCT116 clones with stable expression of the IMS-Prdx5 protein. An alternative approach would have been to use an inducible promoter system, which would have allowed us to grow the tumor xenografts in the absence of IMS-Prdx5 expression and then to administer the activator once the tumors were established. Such an approach would have provided information about whether IMS-Prdx5 expression in an established tumor would cause regression.

## Experimental procedures

### Cell culture

All cells were cultured in a humidified incubator maintained at 37 °C and 5% CO_2_. 143B and 143B(A) human osteosarcoma cells were cultured in modified Eagle medium (Cellgro). HCT116 human colorectal tumor cells were cultured in McCoy’s 5A medium (Cellgro). All cells were acquired from the American Type Culture Collection and cultured according to the vendor’s recommendations. Stable cell lines were obtained *via* Lipofectamine 2000 (Invitrogen) transfection with mammalian expression vectors. Clonal selection of cells was achieved by supplementing media with either G418 (Cellgro), Zeocin (Invitrogen), or Puromycin (Thermo Fisher Scientific) selection agents, depending on the expression vector used.

### Generation of cell lines with stable expression of nondegradable HIF-1α and HIF-2α

Retroviral vectors encoding nondegradable forms of either HIF1α or HIF2α (26) were obtained from Addgene (HA-HIF1alpha-P402A/564A-pBABE-puro and HA-HIF2alpha-P405A/P531A-pBABE-puro (Addgene plasmids # 19005 and # 19006). These were transiently transfected into 293T cells along with a packaging plasmid Gag/Pol and an envelope plasmid VSV-G using Lipofectamine 2000 (Invitrogen). After 48 h, media containing retrovirus from these constructs was collected and filtered through a 0.45 μm filter. That media (with the addition of 0.1% polybrene) was then used to infect HCT116 Clone 70 cells by adding 1 ml of media to each well of a 6-well plate that had been plated on the previous day. The 6-well plate was centrifuged at 2500 rpm at room temperature for 90 min. The cells were then incubated in a 5% CO_2_ incubator for 8 h, after which an additional 1 ml of normal media was added and the cells were returned to the 5% CO_2_ incubator. After 48 h the infected cells were switched to selection media (McCoy’s 5A + 10% fetal bovine serum (FBS), 1% penicillin/streptomycin containing puromycin 2 μg/ml).

The nondegradable-HIF1α or HIF2α-expressing HCT116 Clone 70 cells then underwent single cell clonal expansion. Briefly, the cells were plated onto multiple 10-cm tissue culture dishes at an extreme dilution (1:1000–1:5000) and individual, isolated cells were harvested and maintained in a 5% CO_2_ incubator. The colonies were then expanded in selection media until they reached confluence on 10-cm dishes. Clones were then screened by Western blotting.

### IMS targeting of Prdx5

Second mitochondria-derived activator of caspases or Diablo homolog (smac/Diablo) IMS targeting sequence was PCR-amplified from mouse cDNA using the following primers with *XhoI/HindIII* restriction enzyme sites: Forward primer: 5′ GAT CTC GAG ATG GCG GCT CTG AGA AGT 3′; Reverse Primer: 5′ GGC AAG CTT AAT AGG AAC CGC ACA 3′. The roGFP, first described by Remington *et al.* (49) was PCR-amplified from the cytosolic-targeted roGFP protein in the VQ Ad5CMV K-NpA adenoviral shuttle vector (ViraQuest Inc) described (20) using the following primers with *HindIII/NotI* restriction enzyme sites: Forward primer: 5′ GAG AAG CTT ATG GTG AGC AAG GGC GAG 3′; Reverse primer: 5′ TAT GCG GCC GCT TAA CTT GTA CAG CTC GTC 3′. The smac/Diablo targeting sequence was ligated to the roGFP construct and inserted into the VQ Ad5CMV K-NpA shuttle vector to create a recombinant adenovirus for use with our cells. Full length Prdx5 was PCR-amplified from human cDNA using the following primers with HindIII/EcoRI restriction enzyme sites and a HA tag at the C-terminus end of the protein: Forward primer: 5′ GAG AAG CTT ATG GCC CCA ATC AAG GTG GGA GAT GCC 3′; Reverse primer: 5′ GGC GAA TTC TCA AGC GTA ATC TGG AAC ATC GTA TGG GTA GAG CTG TGA GAT GAT ATT 3′. For use with 143B cells, the smac/Diablo targeting sequence was ligated to the Prdx5 construct and inserted into the pcDNA3.1(−)/Myc-His B mammalian expression vector (Invitrogen). For use with 143B(A) cells, the IMS-Prdx5 construct was inserted into the pcDNA3.1/Zeo(−) mammalian expression vector (Invitrogen).

### Mutagenesis

The QuikChange Site-Directed Mutagenesis Kit (Stratagene) was used according to manufacturer’s instructions to mutate the peroxidatic Cysteine residue of Prdx5 to an Alanine residue. The following primers were used with the pcDNA3.1/Zeo(−) IMS-Prdx5 construct: Sense primer: 5′-GGC CTT CAC CCC TGG AGC TTC CAA GAC ACA CCT G-3′, Antisense primer: 5′-CAG GTG TGT CTT GGA AGC TCC AGG GGT GAA GGC C-3′.

### Immunostaining and confocal microscopy

Cells were plated on collagen-coated coverslips. Cells were fixed in 3% formaldehyde and 0.25% glutaraldehyde in PBS for 15 min and washed in PBS three times. Cells were permeabilized in 0.1% Triton X-100 for 5 min, exposed to three 5 min washes in 0.5 mg/ml sodium borohydride in PBS to reduce the aldehyde groups, and blocked in 1% natural goat serum in PBS for 1 h. Cells were then incubated in primary antibody in 1% natural goat serum for 1 h followed by incubation in secondary antibody for 1 h prior to affixing the coverslips to slides for imaging. Antibodies used were as follows: High affinity anti-HA (Roche, #11867423001, 1:200 dilution), Alexa Fluor 488 (Invitrogen, 1:400 dilution), and Alexa Fluor 568 (Invitrogen, 1:400 dilution). PBS washes followed each step. Confocal images were obtained using a Zeiss LSM 510 META laser scanning confocal microscope with a 40× oil immersion lens and the Zeiss LSM imaging software (Carl Zeiss MicroImaging) (https://www.zeiss.com/microscopy/en/products/software.html#highlights).

### Oxygen consumption

143B cells were trypsinized and resuspended in full media at a concentration of 2 million cells/ml. The respiration rate of 143B cells was measured with a Clark-type oxygen electrode (Oxygen electrode Units DW1; Hansatech Instruments) at 37 °C.

### Hypoxia

Cells were placed in an environmental hypoxia chamber (Coy Laboratory Products) maintained at 1.5% oxygen, 5% carbon dioxide, and the balance nitrogen. For Western blot experiments, pre-equilibrated hypoxic media was added to cells at the start of the experiment and tissue culture dishes were gently rocked on an oscillating platform prior to cell lysate collection. Cells for ROS and proliferation experiments were placed in a static incubator inside an environmental chamber maintained at 1.5% O_2_/5% CO_2_ at 37 °C.

### Epifluorescence microscopy

Coverslips were placed into a flow-through chamber consisting of two coverslips separated by a stainless steel spacer ring. In the chamber, cells were superperfused with a balanced salt solution consisting of 117 mM NaCl, 4 mM KCl, 18 mM NaHCO_3_, 0.76 mM MgSO_4_, 1 mM NaH_2_PO_4_, 1.21 mM CaCl_2_, and 5.6 mM glucose and bubbled with O_2_-CO_2_-N_2_ gas mixtures at 37 °C in a water-jacketed column. Normoxic cells were bubbled with 5% CO_2_, 21% O_2_, and the balance N_2_. For hypoxia, balanced salt solution was bubbled with 5% CO_2_, 1.5% O_2_, and the balance N_2_.

### Oxidant signaling measurements

Cells were plated on collagen-coated 25-mm glass coverslips and infected with virus for 24 h to induce expression of targeted roGFP, prior to placing them in normoxia or hypoxia for 12 to 30 h. For roGFP live cell epifluorescence measurements, excitation wavelengths of 400 and 485 nm were used and fluorescence was detected at 535 nm using a 16-bit cooled charge-coupled device detector. Regions of interest were outlined in the 485/400 ratiometric images produced by the Metafluor software (Molecular Devices) (https://support.moleculardevices.com/s/article/MetaMorph-Software-installation-files) and images were acquired at 1-min intervals for the duration of the experiment. A stable baseline was monitored for 5 min and averaged prior to fully reducing the probe with 1 mM DTT and then fully oxidizing the probe with 1 mM *tert*-butyl hydroperoxide. This allowed calculation of the cellular redox status under baseline conditions. Individual cells were identified as regions of interest and the percent oxidation was calculated by averaging these regions after calculating their redox state. All results were calculated as a percent oxidation of the respective roGFP probe, using the fully oxidized and fully reduced values as reference values (49, 50). Notably, roGFP oxidation does not provide a direct measure of H_2_O_2_ levels. Rather, it provides a readout of the thiol redox status in the compartment where it is expressed. Increases in H_2_O_2_ production cause a shift in the oxidation status of the glutathione pool, which produces a corresponding increase in the oxidation of the roGFP pool.

### Antibodies and Western blotting

Cells were lysed in a buffer consisting of Tris–HCl pH 7.4 (50 mM), NaCl (150 mM), Triton X-100 (1%), EDTA (2 mM), β-glycerophosphate (40 mM), PMSF (1 mM), NaF (10 mM), sodium orthovanadate (250 μM), and a protease inhibitor cocktail (Roche). Hypoxic samples were lysed inside the hypoxia chamber to prevent sample reoxygenation. Lysates were separated on SDS-polyacrylamide gels and transferred to nitrocellulose membranes that were blotted with primary antibodies. Blots were further incubated with secondary horseradish peroxidase-conjugated antibodies (Cell Signaling) and stained with enhanced chemiluminescence reagent (Amersham). Chemiluminescence was detected on either film or using a ChemiDoc XRS+ System (Bio-Rad) and quantified using either Image J (https://imagej.nih.gov/ij/download.html) or the Bio-Rad Image Lab Software (https://www.bio-rad.com/en-us/product/image-lab-software?ID=KRE6P5E8Z). The primary antibodies used were as follows: β-actin (Abcam, #ab6276, 1:10,000 dilution), HIF-1α (BD Biosciences, #610958, 1:1000 dilution), Hydroxy-HIF-1α (Pro564) (Cell Signaling, #3434, 1:1000 dilution), anti-HA tag (Abcam #ab9110, 1:1000 dilution), HIF-2α (Novus Biological, #NB-100-122, 1:1000 dilution), and PDHK1 (Cell Signaling, #C47H1, 1:1000 dilution).

### *In vitro* cell proliferation assay

For each cell line analyzed, cells were plated onto 10 cm dishes the day before the experiment began. On each of the next 7 days, a dish from each cell line was dissociated with 0.05% trypsin, pelleted by centrifugation, and resuspended in 1 ml of Dulbecco's modified Eagle's medium media. The total number of cells on each dish was then determined using a Bio-Rad TC20 automated cell counter. The resuspended cells were recounted four times and the total cell count for each day was taken as the average of these counts. This process was carried out six times. The resulting cell growth data were normalized to the growth rate of WT HCT116 cells. Overall data were analyzed by two-way ANOVA for repeated measures; when statistical differences were detected, post hoc analysis by Tukey’s multiple comparison test were used to explore individual comparisons.

### Tumor xenografts

Control and IMS-Prdx5 cells were resuspended in PBS at a concentration of 5 × 10^6^ cells per 100 μl. Athymic nude mice (5-week old) were injected in one flank with 100 μl PBS containing control cells and in their other flank with 100 μl of IMS-Prdx5 expressing cells. All mice received the same tumor cell dose. Tumor sizes were monitored repeatedly using calipers for 20 days, beginning at ∼5 days after inoculation. All tumors were harvested at the same time, which was ∼25 days after inoculation. Tumor volume was calculated using the formula Volume = (Length × Width^2^)/2. Tumors were then excised, weighed, and processed for Western blot analysis. All mouse procedures were carried out after institutional review and approval by the Animal Care and Use Committee at Northwestern University.

### Soft agar assay

Colony formation on soft agar was carried out as described (51). Briefly a 1% base agar medium was prepared with 1% noble agar mixed with McCoy’s 5A medium containing 10% FBS and 1% penicillin/streptomycin plus other antibiotics used for selection. An aliquot (1 ml) of this mixture was transferred to each well of a 6-well plate and allowed to cool for 30 min at room temperature. The cells to be cultured were trypsinized, counted, and seeded at 5 × 10^3^ cells/well in a 0.6% upper agar layer also containing noble agar mixed with McCoy’s 5A medium containing 10% FBS and 1% penicillin/streptomycin plus other antibiotics. This layer was allowed to solidify for 30 min at room temperature. Next, 100 μl of culture media was added above the top agar layer to prevent desiccation (this was reapplied every 3–4 days). The plates were incubated at 37 °C under room air containing 5% CO_2_ for 21 days. To stain the cells and count the colonies, 200 μl of nitroblue tetrazolium chloride solution was added to each well and the plates were incubated overnight in a 37 °C incubator. Once the colonies were stained, they were photographed and counted using Image J analysis software.

### RNA isolation and real-time quantitative RT-PCR analysis

An RNA purification kit (RNAeasy Plus Mini-Kit, Qiagen) was used to isolate total RNA from tumors harvested from nude mice (injected subcutaneously with control A and IMS-Prdx5 clone 32 cells) as well as from HCT116 Control P and IMS-Prdx5 clone 70 cells that were grown in either normoxia or hypoxia (1.5% nitrogen) for up to 4 h prior to isolation. cDNA was prepared from the RNA using the iScript Reverse Transcription Supermix (Bio-Rad) and real-time RT-PCR (qPCR) was performed using the iQ SYBR Green PCR Supermix (Bio-Rad). mRNA expression of PDHK1 and the reference gene β-actin were measured on a CFX96 qPCR cycler (Bio-Rad). Primers used were: PDHK1 Forward: GGCTGGTTTTGGTTATGGATTG, PDHK1 Reverse: CTGGGAGTCTTTCTATTGAGTCTG, β-actin Forward: ATAGCACAGCCTGGATAGCAACGTAC, and β-actin Reverse: CACCTTCTACAATGAGCTGCGTGTG. Gene regulation of PDHK1 was quantitated by the 2^−ΔΔCt^ method with normalization to the β-actin level. Results are expressed as a mean value ± SEM of either the PDHK1/β-actin fold change or the log2 fold change.

To quantify the relative expression levels of IMS-Prdx5 compared to endogenous Prdx5 we performed RT-qPCR. Total RNA was isolated from HCT116 WT cells and IMS-Prdx5 clone 70 cells and cDNA was prepared and RT-qPCRs were performed as described above. mRNA expression of IMS-Prdx5 and native Prdx5 and the reference gene β-actin were measured on a CFX96 qPCR cycler (Bio-Rad). Primers used were as follows: Prdx5 Forward: GATTCGCTGGTGTCCATCTT (used for both native Prdx5 and IMS-Prdx5 qPCR reactions), Prdx5 Reverse: ACATTCAGGGCCTTCACTATG, IMS-Prdx5-HA Reverse: CTGGAACATCGTATGGGTAGAG β-actin Forward: ATAGCACAGCCTGGATAGCAACGTAC, and β-actin Reverse: CACCTTCTACAATGAGCTGCGTGTG. Gene regulation of IMS-Prdx5 and native Prdx5 was quantified by the 2^−ΔΔCt^method with normalization to the β-actin level. Results are expressed as a mean value ± SD of Prdx5/β-actin fold change and IMS-Prdx5/β-actin fold change or the log2-fold change.

### Statistics

The Grubbs outlier test was performed to determine if any data points were to be excluded from analysis. ANOVA was used to identify differences between multiple experimental groups. Newman-Keuls post hoc analysis or Tukey’s multiple comparison test was used to determine significance involving multiple groups. A student’s *t* test was used when only two groups were being compared. Significance was accepted at the *p* ≤ 0.05 level.

## Data availability

All data are included in the article.

No datasets have been deposited into a repository.

No proprietary software code was used for the analysis.

There are no exceptions or limitations to the sharing of data, materials, or software.

## Supporting information

This article contains supporting information.

## Conflict of interest

The authors declare that they have no conflict of interest with the contents of this article.

## Figures and Tables

**Supporting Figure S2:**